# Factors and prediction of carbon emissions based on PSO-BP neural network model under the development of digital economy

**DOI:** 10.1371/journal.pone.0322703

**Published:** 2025-05-08

**Authors:** Wen Liu, Yang Lan, Mi Li, Wen Tu

**Affiliations:** 1 School of Business Administration, Moutai Institute, Zunyi, China; 2 TVES Laboratory, The University of Lille, Lille, France; 3 School of General Education, Moutai Institute, Zunyi, China; University of Oklahoma, UNITED STATES OF AMERICA

## Abstract

The Yellow River Economic Belt, where the degree of digital economy development is uneven, is the first research object used in this study. It then suggests a way to measure the degree of digital economy development and carbon emissions in order to address the problem of effectively controlling carbon emissions in the rapidly developing digital economy. Finally, a genetic method is presented to further enhance the backpropagation neural network model’s update process, which was improved utilizing the particle swarm optimization technique. According to the findings, this research identified three primary elements: digital industrialization, digital finance, and digital ecological environment. According to the findings, this research identified three primary elements: digital industrialization, digital finance, and digital ecological environment. With the use of digital technology, the digital ecological environment fosters a peaceful coexistence between people and the natural world. In addition to encouraging the advancement of digital technology, it may also help to integrate digital transformation and green development. The use of digital technology in ecological environment governance can assist accomplish sustainable development goals, improve resource allocation, and encourage intelligent and green production and life. In order to change conventional financial service models, the financial sector known as “digital finance” makes use of digital technologies and data components. It has the potential to be very important in encouraging industrial upgrading and propelling the growth of new industries. Additionally, the whole credit structure of the industrial chain may be improved by digital credit and risk management, which will support the economic structure’s optimization. The use of digital technology to a variety of sectors, encouraging their digital transformation and modernization, is known as digital industrialization. It is a key component of a contemporary industrial system that may drive new industries and formats, support the intelligent and information-based transformation of established industries, and improve the economic structure. At the same time, the associated carbon emissions dropped by 0.0439 units for every unit rise in the study area’s digital economy’s degree of growth. The region’s overall population, energy consumption, sophisticated industrial structure, and industrial structure rationalization all positively promote carbon emissions, whilst other variables have the opposite impact. The final study approach had the highest predictive performance, with a high goodness of fit of 0.9936 and an average absolute error of 16.971. The aforementioned study results demonstrate that the methodology can effectively evaluate the level of carbon emissions and the development of the digital economy across different regions and provide targeted solutions to lower carbon emissions in line with local conditions, thus fostering the vibrancy of the digital economy.

## Introduction

In the current era, the Digital Economy (DE) has driven profound changes in production, lifestyle, and national governance. This provides strong support for China to complete its Dual Carbon Goals (DCGs), further transform its economic development mode, and achieve High-quality Economic Development (HQED). It also injects new momentum into the green development of the economy [1–3]. The widespread application of digital technology in business has given rise to various new formats such as mobile payments and the sharing economy, reducing energy consumption and Carbon Emissions (CE) in multiple stages of production and consumption [4–5]. Additionally, the usage of digital technology in government services has had a profound impact on the governance model of the government. It improves the efficiency of government management services, and is essential in reducing CE in the development of the real economy [6–7]. This is because digital technology can improve the efficiency of green technology research and resource allocation, thereby reducing costs and carbon emissions. At the same time, it can promote the transformation and upgrading of traditional industries, give birth to new modes and formats, and reduce carbon emission intensity. In addition, digital technology can improve resource utilization by optimizing resource allocation and generating collaborative optimization effects, making production and transportation processes more rational and efficient, reducing resource waste, and suppressing carbon emission intensity. However, as the DE industry becomes a primary player in CE, the Digital Economy Development (DED) will reduce the CE intensity. DED is a new economic form that relies on key elements of data resources, modern information networks as the main carrier, and the integration of information and communication technology and the digital transformation of all factors as important driving forces. Therefore, how to deal with the contradiction between the DED level and CE is current focus. Numerous scholars have researched and explored this matter. Y. Liu et al. selected data from non-financial listed companies in the Shanghai and Shenzhen A-share markets from 2011 to 2018 to investigate whether the China’s DED would have a profound effect in the healthy development of enterprises. They found that digital finance was able to encourage companies to lower their financialization level, which in turn has a great impact on their business performance [8]. Q. He et al. aimed to explore the role of digital inclusive finance and digital technology innovation in achieving low-carbon economic growth in various countries. Therefore, the study analyzed the impact of digital inclusive finance on carbon emission intensity from the perspectives of base effect and scale effect. The research results showed that digital inclusive finance can have a U-shaped impact on carbon emission intensity by enhancing digital technology innovation. This indicates that digital inclusive finance can promote the innovation of digital technology in enterprises and effectively reduce carbon emission levels [9]. R. The aim of Nepal et al. is to explore the impact of international green financing on the low-carbon energy transition of developing countries, as well as the regulatory role of the digital economy in it. The experimental results show that the development of digital technology can promote green financing in the digital economy and provide positive regulatory effects, which is conducive to promoting low-carbon energy transition in various countries. This indicates that green financing can provide financial support for projects in areas such as clean energy and green buildings. For example, by supporting clean energy projects such as wind and solar energy, greenhouse gas emissions can be reduced. By using digital technology and energy-saving materials in green buildings, carbon emissions during the construction process can be reduced [10]. X. Guo et al. chose some factors, including Per Capita Gross Domestic Product (PC-GDP), and CE coefficient etc., for analysis to achieve the DCG as soon as possible. The impact of PC-GDP on CE would gradually decrease in the future, and for certain industries, reducing energy intensity was beneficial for reducing CE [11]. T. Cathy believed that although atmospheric observations could quantify anthropogenic CE, the variability of net-land carbon exchange delayed the detection of carbon change. Therefore, this study improved the understanding of this variability and allowed for earlier detection of CE changes [12]. K. Dong et al. aimed to examine the relationship between Natural Gas Import Safety (NGIS) and CE, and therefore designed a comprehensive index to measure the NGIS level of 30 countries from 2004 to 2019. A panel data model was constructed to estimate the NGIS effect on CE. Estimates suggested that increasing NGIS could indeed reduce global CE [13]. E. J. Johnson et al. proposed a personal emission-related estimation method to explore the factors that greatly affect CE. This method has been inaccurate in evaluating the CE of behavior, enterprises, and industries, which may be due to a lack of information or professional knowledge [14].

Based on the above content, it can be concluded that current research mainly focuses on the development of the digital economy and carbon emissions. However, existing research lacks quantitative analysis of the impact of the digital economy on carbon emissions and intelligent prediction methods. To promote the sustainable development of the digital economy and apply intelligent technology to carbon emission prediction, this study analyzes the impact pathways of carbon emissions under the development of the digital economy, and designs methods for measuring the level of digital economy development and carbon emissions. Finally, Particle Swarm Optimization (PSO) and Genetic Algorithm (GA) were used to optimize the Back Propagation (BP) neural network, resulting in a Combination Improvement Prediction (CIP) model. The research aims to explore in depth the influencing factors and degree of regional DED on CE, and then develop targeted control plans for CE according to local conditions, allocate limited resources reasonably, promote the rapid DED, and achieve the DCGs as soon as possible. The novelty of the research mainly lies in two aspects. On the one hand, it deeply analyzes the relationship between digital economy and carbon emissions from the perspective of both economic and environmental benefits, in order to achieve a win-win situation between economic development and ecological environment protection; On the other hand, advanced digital technologies are applied in carbon emission prediction, and CIP models are designed to achieve more accurate carbon emission prediction and provide more valuable information. The contribution of the research lies in providing scientific basis and decision support for achieving the dual carbon goals in different regions, enriching the impact mechanism of the digital economy on carbon emissions, and providing new theoretical support for promoting regional green and high-quality development. The research structure mainly consists of the following three parts. The first part introduces the influencing factors and pathways of carbon emissions under the development of the digital economy, proposes regional digital economy level and carbon emission measurement methods, and designs a CIP model to predict carbon emissions. The second part is to explore the level of digital economy development and carbon emission measurement results in the research area, and based on this, analyze the impact and effectiveness of carbon emissions, and finally analyze the effectiveness and feasibility of the prediction model. The third part is a summary of the research results.

## 1 Materials and methods

Firstly, the influencing factors and pathways of CE under DED are introduced. Then, the DED level and CE measurement method in the study area are proposed. Finally, a CIP model is designed to test the CE quantity in the study area.

### 1.1 The influencing factors of carbon emissions under the development of digital economy

**Fig 1:**
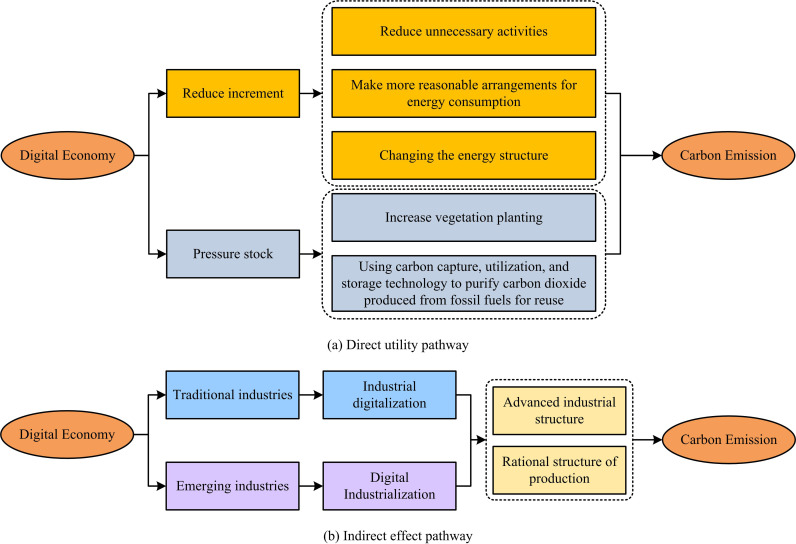
The mechanisms determining the paths of CE.

The Chinese economy is facing triple pressures of demand contraction, supply shock, and weakened expectations. How to seize opportunities and promote HQED with new impetus has become a key concern for many researchers [15–16]. Moreover, at a critical moment of deep global economic adjustment, competition among countries around the world is intensifying, and the DE born from the new round of technological revolution has become the main battlefield for major powers to seize the high ground. Vigorously developing the DE to promote HQED and low-carbon economy is not only an opportunity given by the times, but also faces numerous challenges [17–19]. In the context of global climate change and increasingly severe environmental pollution, the DCG has become a shared responsibility and mission of all countries. China has actively responded to and implemented the dual carbon policy, and further transformed its economic development mode through the DE, injecting new momentum into the green economy development and promoting the comprehensive green transformation of the economy and society [20–22]. The influencing factors of CE under DED are divided into two pathways: direct effects and indirect effects, as shown in Fig 1.

Figures 1(a) and (b) correspond to the direct and indirect effects of the influencing factors on CE under DED. In terms of direct effects, the DE is mainly achieved through reducing increment and suppressing stock. The former is mainly achieved through the following three ways: Firstly, reducing unnecessary activities; The second is to make more reasonable arrangements for energy consumption, making economic activities more efficient; The third is to change the energy structure, making the CE per unit of energy consumption lower [23–25]. The latter has two main ideas: One is to increase vegetation planting and absorb carbon dioxide from the air through photosynthesis of plants; The second is to use carbon capture, utilization, and storage technology to purify the carbon dioxide produced by fossil fuels for reuse, or to compress the generated carbon dioxide into liquid form to prevent it from directly entering the atmosphere [26–28]. The indirect effects are analyzed from the demand side. On the one hand, low-carbon technology is currently very expensive and requires significant funding for enterprise transformation. Therefore, traditional enterprises can be encouraged to develop digital low-carbon technologies through green finance or sustainable finance. On the other hand, it is to industrialize digital technology and guide people’s behavior through small policy designs. For example, Ant Forest’s attempt to digitize people’s environmental behaviors and present them in a virtual way through tree planting, ultimately obtaining real trees as rewards.

### 1.2 The level of digital economy and carbon emission measurement methods in the research region

This paper takes the Yellow River Economic Belt (YREB) as the research object, and promoting its coordinated development has become an important part of promoting ecological environment protection in the Yellow River Basin.It is located in the area along the Yellow River and is mainly an economic belt formed by the distribution of areas along the Yellow River, including 9 provinces and regions including Shandong, Henan, Shanxi, Shaanxi, Inner Mongolia, Ningxia, Gansu, Qinghai, and Xinjiang, denoted as S1 ~ S9 respectively. The coverage area is 1.3 million km^2^, as shown in Table 1.

**Table 1:** Composition of the research area.

Province in China	Number
Shandong	S1
Henan	S2
Shanxi	S3
Shaanxi	S4
Inner Mongolia	S5
Ningxia	S6
Gansu Province	S7
Qinghai	S8
Xinjiang	S9

**Fig 2:**
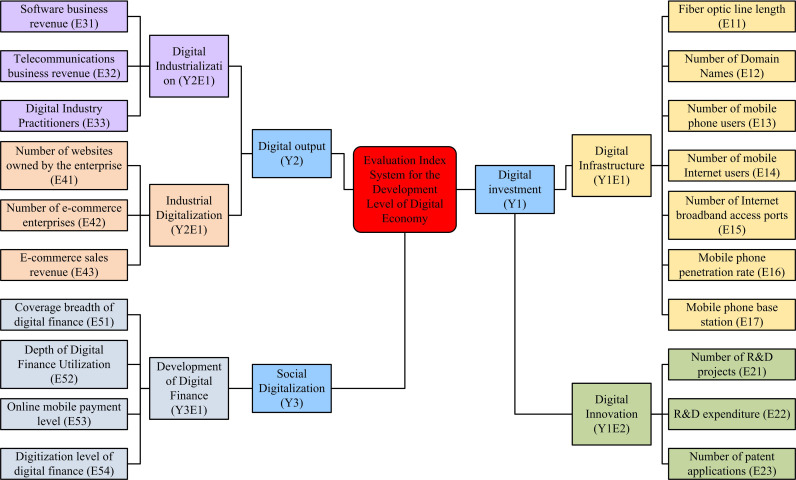
EIS for the regional DED level.

Among them, PC-GDP and industrial structure are indicators for judging the development status of regional economy. The PC-GDP of the research area has long been significantly lower than the national level. However, if analyzed by region, the PC-GDP corresponding to S1 and S5 provinces is higher than the national average. Before 2015, the proportion of the secondary industry in the research area was the highest, and the development of the tertiary industry was relatively slow. However, after that, the industrial structure of the region is consistent with that of the whole country, mainly relying on the tertiary industry. After introducing the basic situation of the research area, an Evaluation Index System (EIS) can be established to measure the level of DED and CE. In the construction of EIS, the following principles need to be followed: comparability and quantifiability, systematicity and typicality. The same indicator needs to have the same evaluation scale for all evaluation objects, and the indicators need to be quantified. There is a logical relationship between the indicators. After comprehensive consideration, the DED level EIS of the study area can be obtained, as shown in Fig 2.

In Fig 2, the primary indicators include Y1, Y2, and Y3. The secondary indicators include digital infrastructure (Y1E1), digital innovation (Y1E2), digital industrialization (Y2E1), industrial digitization (Y2E2), and digital financial development (Y3E1). There is a total of 20 third level indicators. The digital ecological environment refers to the path and mode of ecological civilization construction through digital technology innovation, in order to promote the coordinated development of ecological civilization construction, digital economy, digital society, etc. It emphasizes the value of digital technology innovation, broadens digital application scenarios, leads green development with digitalization, and drives industrial digital transformation. Digital finance is the product of the deep integration of digital technology and financial services. Through Internet technology, information communication technology and digital technology, it can effectively reduce transaction costs, promote market competition, and solve the problem of information asymmetry in financial activities. It can accelerate the flow of funds, information, and data, help accelerate the formation of new quality productivity, and assist enterprises and organizations in more accurate grain production and carbon footprint management through the use of cash data analysis and technological tools, thereby promoting green technology innovation and reducing carbon emission intensity. Digital industrialization is the foundation and driving force of the development of the digital economy. Through technological innovation and application promotion, it constitutes the leading industry for the development of the digital economy. The digital ecological environment enhances the governance capability of the ecological environment through digital means, digital finance reduces carbon emissions by optimizing resource allocation and energy efficiency, and digital industrialization provides basic support to promote the digital transformation of traditional industries. The above evaluation is straightforward. Together, it has promoted the high-quality development of the digital economy and facilitated profound changes in the way society produces and governs. There are two main ways to determine the weight of indicators, namely objective weighting method and subjective weighting method. Previous research results have shown that the Entropy Method (EM) is the most widely used objective weighting method, which can eliminate the result bias caused by subjective assignment. Due to the certain correlation between EIS, using EM alone will lack subjective judgment information. Therefore, this study combines EM with global Principal Component Analysis (PCA) to enhance the credibility of the evaluation results. The process of the global PCA method is as follows. Firstly, assuming that there are sample individuals and features *a* and b, and the data are represented by $X_{b}$, the data table $X^{n}={\left(x_{ij}\right)}_{a⬝b}$ for year n can be obtained. The data table is summarized by year to obtain new data $X={\left(x_{ij}\right)}_{Na⬝b}$ with temporal characteristics. Secondly, the data are dimensionless to improve its quality and operability. This study chooses the commonly used Z-score method for processing, and its expression is equation (1).


$$v_{ij}=\frac{x_{ij}-{\bar{x}}_{ij}}{s}$$ (1)

In equation (1), ${\bar{x}}_{ij}$ and s correspond to the mean and standard deviation of the original sequence. Before PCA operation, Kaiser-Meyer-Olkin test (KMO) and Bartlctt test need to be completed. If the former is greater than 0.7 and the latter has a probability value less than 0.05, it means that PCA method can be used. After the verification is completed, the correlation coefficient R, eigenvalues $\zeta_{i}$, and eigenvectors corresponding to the processed matrix V can be calculated. Then, the variance contribution rates of all $\zeta_{i}$ are calculated, and the principal components with $\zeta_{i}$ exceeding 1 are set as the main components. After a series of calculations, the comprehensive score can be obtained. The EM is utilized to decide the dispersion degree of indicators. The greater the dispersion degree indicates better the impact of the corresponding indicators on the comprehensive evaluation. The process of EM first requires normalization of the data, and the specific calculation is given by equation (2).


$$Z_{ij}=\left\{ \begin{array}{cc} & \frac{X_{ij}-\min\left(X_{j}\right)}{\max\left(X_{j}\right)-\min\left(X_{j}\right)}⬝10,\text{ Positive direction}\\ & \frac{\max\left(X_{j}\right)-X_{ij}}{\max\left(X_{j}\right)-\min\left(X_{j}\right)}⬝10,\text{ Negative direction} \end{array}\right.$$ (2)

In equation (2), $\max\left(X_{j}\right)$ and $\min\left(X_{j}\right)$ are the Max and Min of the data. The expression for calculating the information entropy of the indicator is equation (3).


$$E_{j}=-\frac{1}{\ln aM}\sum_{m=1}^{M}\sum_{i=1}^{b}z_{ij}^{m}\ln z_{ij}^{m}$$ (3)

The calculation of the information utility value $h_{j}$ and weight coefficient $w_{j}$ corresponding to the final indicator is equation (4).


$$\left\{ \begin{array}{cc} & h_{j}=1-E_{j}\\ & w_{j}=\frac{h_{j}}{b-\sum_{j=1}^{b}h_{j}} \end{array}\right.$$ (4)

The CE measurement method for the research area was analyzed using the data from 2016 to 2023 in the provincial level CE inventory of China’s carbon accounting database, and the accounting was conducted using the methods of the United Nations Intergovernmental Panel on Climate Change. Firstly, it is necessary to determine the emission source. The types of emission sources vary greatly, such as oil combustion, coal combustion, human respiration, etc. Then we need to determine the CE factor, which is the coefficient of CE dioxide for a certain energy consumption process. Finally, the corresponding CE amount can be obtained by multiplying the usage of the emission source by the CE factor of the emission source. Based on the above data and analysis methods, solid data support can be laid for the prediction of CE quantity in the subsequent research area.

### 1.3 Carbon emission prediction method based on PSO-BP neural network model

**Fig 3:**
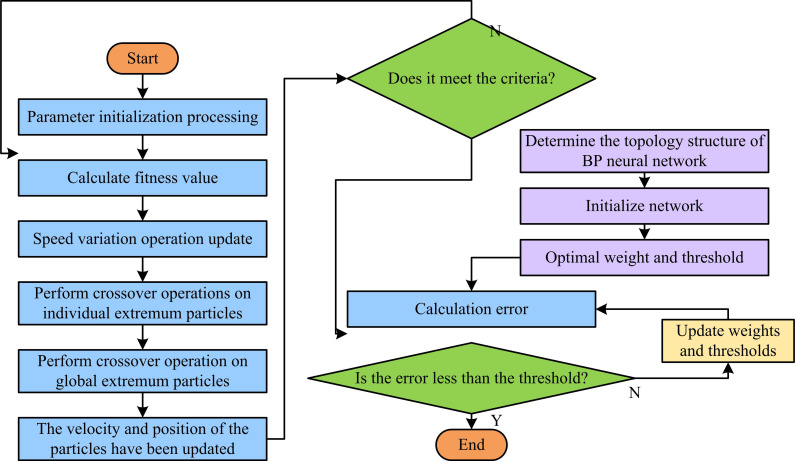
Specific process diagram of CIP model.

To further predict and analyze the CE quantity in the research area, this study introduces PSO and GA for optimization based on BP network, and obtains a CIP model of PSO-BP network improved by GA. Fig 3 shows the flowchart of this model.

In Fig 3, the parameters of PSO and BP are initialized first, and then the fitness values are calculated. At the same time, individual extremum and population extremum are screened. Then, the mutation and crossover operations are completed, and the information corresponding to the velocity and position of the particles is updated. The above operation is repeated until the optimal fitness value is obtained, while updating the weights and thresholds to obtain the initial values of BP. Finally, training according to the determined learning method until the output conditions are met, and the final prediction result can be obtained. The specific design is as follows. Firstly, the fitness function uses the Sum of Squared Errors (SSE), and the calculation is equation (5).


$$fit={\sum_{i=1}^{m}\left(v_{p}-v_{t}\right)}^{2}$$ (5)

In equation (5), $v_{p}$ and $v_{t}$ are the expected and actual outputs. Secondly, in the design of genetic operators, the process of individual speed variation is equation (6).


$$\left\{ \begin{array}{cc} & SD_{i}\left(k+1\right)=\left\{ \begin{array}{cc} & SD_{i}(k)+f(t)\left[SD_{i}(k)-SD_{\max}\right],\psi_{1}\geq 0.5\\ & SD_{i}(k)+f(t)\left[SD_{\min}-SD_{i}(k)\right],\psi_{2}<0.5 \end{array}\right.\\ & f(t)=\psi_{3}\frac{1-t}{t_{\max}} \end{array}\right.$$ (6)

The Max and Min particle velocities corresponding to $SD_{\max}$ and $SD_{\min}$ in equation (6). t and $t_{\max}$ are the current iteration times and Max evolution times. $\psi_{1}$ and $\psi_{3}$ are both random numbers from 0 to 1. The process of individual positional variation is equation (7).


$$\left\{ \begin{array}{cc} & S_{i}\left(k+1\right)=\left\{ \begin{array}{cc} & S_{i}(k)+f(t)\left[S_{i}(k)-S_{\max}\right],\psi_{1}\geq 0.5\\ & S_{i}(k)+f(t)\left[S_{\min}-S_{i}(k)\right],\psi_{2}<0.5 \end{array}\right.\\ & f(t)=\psi_{3}\frac{1-t}{t_{\max}} \end{array}\right.$$ (7)

The upper and lower bounds of the particle positions corresponding to $S_{\max}$ and $S_{\min}$ in equation (7) represent random numbers from 0 to 1. In the design of crossover operators, the velocity crossover process is equation (8).


$$\left\{ \begin{array}{cc} & SD_{i}(k+1)=\tau_{1}SD_{i}(k)+SD_{j}(k)(1-\tau_{1})\\ & SD_{j}(k+1)=\tau_{1}SD_{j}(k)+SD_{i}(k)(1-\tau_{1}) \end{array}\right.$$ (8)

In equation (8), $\tau_{1}$ is a random number from 0 to 1. The process of position crossing operation is equation (9).


$$\left\{ \begin{array}{cc} & S_{i}(k+1)=\tau_{2}S_{i}(k)+S_{j}(k)(1-\tau_{2})\\ & S_{j}(k+1)=\tau_{2}S_{j}(k)+S_{i}(k)(1-\tau_{2}) \end{array}\right.$$ (9)

In equation (9), $\tau_{2}$ is a random number from 0 to 1. In the selection operator, the expression for individual selection probability is equation (10).


$$P_{S}=\frac{1/E\;}{\sum {\mathrm{\backslash nolimits}}_{1}^{M}\left(1/E\;\right)}$$ (10)

In the parameter design of PSO algorithm, it is mainly divided into inertia weight ω and acceleration factors $c_{1}$ and $c_{2}$. The former is calculated through the strategy of decreasing inertia weight, as shown in equation (11).


$$\omega =\omega_{\max}-\frac{t\left(\omega_{\max}-\omega_{\min}\right)}{t_{\max}}$$ (11)

In equation (11), $\omega_{\max}$ and $\omega_{\min}$ correspond to the Max and Min values of ω. In previous experimental results, $c_{1}$ and $c_{2}$ values of 1.85 and 2 can effectively improve the stability of the method’s operation. In BP networks, the amount of neurons in the hidden layer is closely related to the accuracy of the model, but determining its quantity is somewhat difficult. Therefore, this study uses mainstream empirical formulas for calculation, such as equation (12).


$$N_{h}=\sqrt{N_{in}+N_{out}}+\alpha$$ (12)

In equation (12), $N_{in}$ and $N_{out}$ are the quantity of nodes in the input and output layers. α is a random number in [1,10]. The initial weights will directly affect the learning performance of the BP network, and usually a Gaussian distribution with mean = 0 and variance = 1 is used for initialization processing. However, this method can cause the output of the activation function to approach 0 or 1 when the initial weight values are large or small, and the corresponding derivative will also tend towards 0 [29–31]. This will cause neurons to become saturated and have minimal impact on other network layers, resulting in minimal changes to the loss function of the entire BP network and slower training speed. To avoid the problem of slow training, this study sets the initial weight range at −1 to 1. The final determination of the learning rate of the BP can complete the design of the CIP model. Normally, the learning rate is selected between 0.01 and 0.8, and the optimal rate can be determined based on debugging experiments.

## 2 Results

To better construct the research area, this study first explores the DED level and CE measurement results of the area, and based on this, analyzes the impact and effectiveness of CE. At last, the effectiveness and feasibility of the prediction model are analyzed.

### 2.1 Development level of digital economy and carbon emission calculation results

The data used come from the statistical yearbooks of various provinces in the study area from 2016 to 2023, the China Regional Digital Research Database, the Digital Economy Industry Special Database, and the China Carbon Accounting Database. Firstly, it is necessary to verify the standardized data to determine whether it can be used with PCA method. The KMO test result is 0.887, and the probability value of the Bartlett test is 0.000, corresponding to a significance level below 0.05. This indicates that there is a relation between the indicators of DED level and EIS, which can be used for subsequent PCA analysis. According to the principle of PCA method, the cumulative contribution rate of variance exceeds 85%, and the components with $\zeta_{i}$ exceeding 1 are taken as principal components, and most of the information of the original indicators will be retained. The PCA results can be obtained from this, as listed in Table 2.

**Table 2:** PCA results.

Principal component		F1	F2	F3
Initial eigenvalue	Total	15.426	3.748	1.027
	Variance percentage/%	66.576	16.743	4.701
	Accumulate	66.576	83.319	88.020
Extract the sum of squared loads	Total	15.426	3.748	1.027
	Variance percentage/%	66.576	16.743	4.701
	Accumulate	66.576	83.319	88.020
Sum of squared rotational loads	Total	13.583	4.577	1.149
	Variance percentage/%	61.824	20.618	5.578
	Accumulate	61.824	82.442	88.020

**Fig 4:**
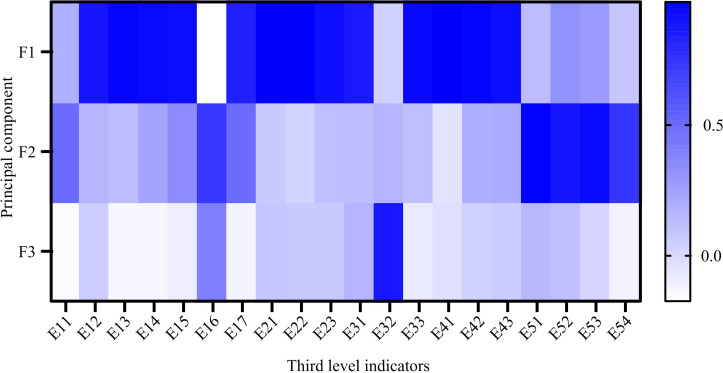
The rotated component matrix results of the third level indicators and principal components.

In Table 2, this study extracts three principal components, F1 ~ F3, with corresponding variance explanatory rates of 66.576%, 16.743%, and 4.701%, and cumulative variance explanatory rate of 88.02%. In the results of the sum of squares of rotational loads, the variance solidification rates of F1 ~ F3 principal components are 61.824%, 20.618%, and 5.578%, respectively, with a cumulative variance explanation rate of 88.02%. Therefore, the rotation component matrix results of the tertiary indicators and principal components are shown in Fig 4.

**Fig 5:**
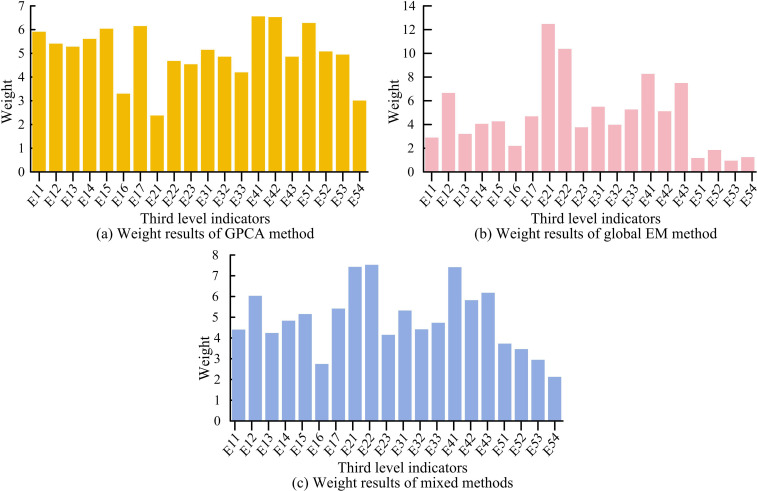
Weight results based on mixed processing method.

In Fig 4, in F1, E12, E15, E17, E21 and other indicators have the highest number of loads. In F2, E51, E52, E53 and other indicators have the highest number of loads. In F3, E16 and E32 have the highest number of loads. F1 ~ F3 respectively represent digital ecological environment, digital finance, and digital industrialization. After processing with the global PCA method and EM method, the weights of the final indicators can be obtained, as shown in Fig 5.

**Fig 6:**
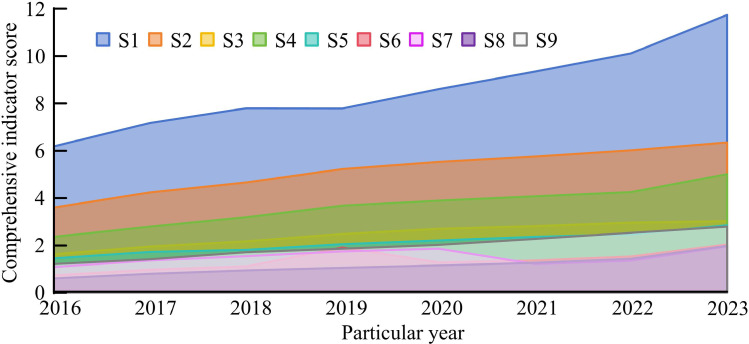
The comprehensive index results of the DED level in various provinces within the research area.

Fig 5(a) shows the weight results of the global PCA method, where the weight values of all indicators are greater than 2, and the weight values of most indicators exceed 4. Fig 5(b) is the results of the global EM. The fluctuation of each indicator is relatively large, with weight values ranging from 1 to 13. Fig 5(c) shows the data of the mixed method. The weight distribution of each indicator is generally balanced, and there are significant differences between the indicators. From the analysis of secondary indicator weights, Y1E1 has the highest weight value at 33.845%, followed by Y2E2 at 19.42%, and Y3E1 has the lowest weight value at 12.275%. This indicates that the foundation of regional DED level depends on infrastructure, and although social digitization has a small weight, it is still an important component of regional DED. Fig 6 shows the comprehensive index of DED levels in each province.

**Fig 7:**
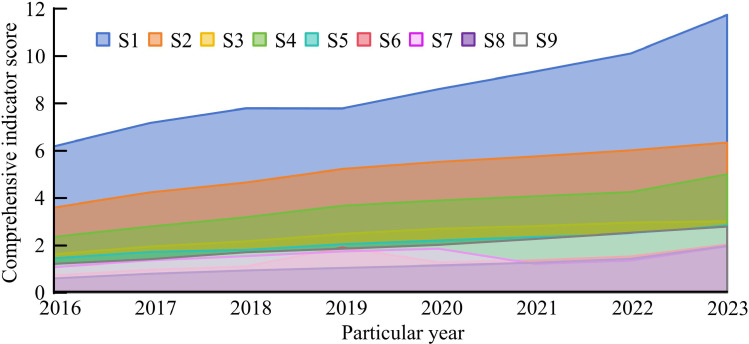
TSE value variation curve within the research area.

In Fig 6 the comprehensive indicator scores of DED levels in S1, S4, and S6 provinces are far ahead in each year, while S8 and S9 provinces have lagged behind throughout the entire process. From the overall trend of change, the Max and Min values of the composite index are 0.6 and 11.75, indicating obvious differences in the DED level within the study area. Overall, with the increase of years, the DED levels of each province have shown a continuous upward trend. Finally, CE measurement is analyzed from two perspectives: Total Carbon Emissions (TCE) and Per Capita Carbon Emissions (PCCE). Among them, the TSE value variation curve within the research area is displayed in Fig 7.

**Fig 8:**
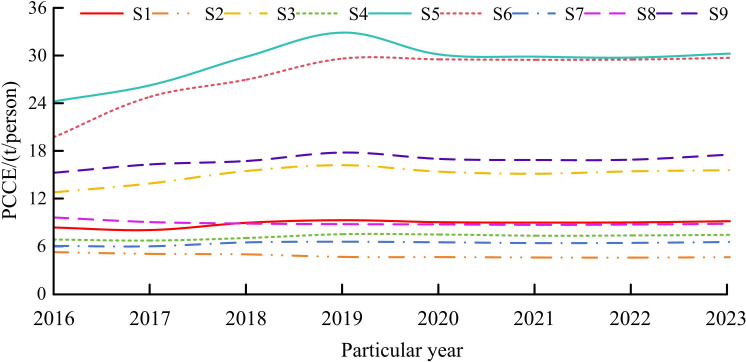
Comparison of PCCE values within the research area.

In Fig 7, overall, each province shows a trend of first growing and then decreasing. This may be since the impact of the COVID-19 in 2020, leading to the reduction of various social and economic production and living activities, resulting in a significant reduction in CE in cities. The TCE values of S1 province are the highest in all years, and the emission control effect in this region is relatively poor. The TSE values of other provinces have significantly decreased after 2020, which may be due not only to the impact of the epidemic, but also to the significant effectiveness of CE control in corresponding regions. After the end of the epidemic, the TSE values of various provinces slightly increases. Fig 8 shows the results of PCCE values within the study area.

In Fig 8, the PCCE values of S5 and S6 provinces are the highest, exceeding 25t/person. Prior to 2019, the growth rate of S5 province was the highest, reaching up to 40.27%. However, in the following period, its growth rate gradually slowed down. The PCCE value in the S2 region is the smallest, indicating that the per capita emission reduction control task in this area is handled well. Overall, the growth rate of most areas in the research region has significantly decreased in recent years. By analyzing the level of DED and the amount of CE in the research area, further exploration can be conducted on the impact and effectiveness of DED on CE in the future.

### 2.2 Analysis of the impact of carbon emissions on the development of digital economy

To further analyze the impact of CE on the research area under DED, this study first uses a Stochastic Impacts by Regression on Population, Influence, and Technology model (STIRPAT) to analyze the emissions of environmental pollutants. In addition, this study optimizes the above model and obtains the optimized model expression, as shown in equation (13).


$$\ln CO_{2}=\alpha +\alpha \ln D+\beta \ln C+\chi_{t}+\varphi_{i}+\varepsilon_{it}$$ (13)

In equation (13), α and β represent the model coefficients and population size. C and D correspond to control variables and DED levels, while $\chi_{t}$, $\varphi_{i}$, and $\varepsilon_{it}$ represent spatiotemporal effects and random error terms. The control variables in the model include Energy Consumption (EC), Total Population of the Region (TPR), Technological Innovation (TI), PC-GDP, Urbanization Level (UL), and Industrial Structure (IS). By taking the logarithm of these ^variables, ln *EC*,^ ln *TPR*^,^ ln *TI*^, ln *PC* − *GDP*, ln *UL*, and ln *IS* can be obtained. In^ addition, this study quantifies the ratio of added value between the tertiary and secondary industries to the advanced level of regional industrial structure, and measures the degree of coordinated development of regional industries through the Rational Structure of Production (RSP). Further, descriptive analysis and stationarity test are conducted in this study, with a sample size of 72. Table 3 lists the related results.

**Table 3:** Descriptive analysis and variable stationarity test results.

Resul t	Variable	ln *CO*_2_	ln *EC*	*D*	*TPR*	ln *TI*	ln *PC *− *GDP*	*UL*	*IS*	*RSP*
	Max	6.853	10.7	7.7	9.3024	16.7	11.2635	0.752	1.82	0.69
		6	426	856		532		6	62	85
Descr	Min	3.857	8.20	0.1	6.2459	10.9	9.8652	0.398	0.70	0.11
iptive		6	16	062		583		5	265	65
Analy	Mean	5.741	9.51	2.2	8.0652	13.7	10.8542	0.597	1.20	0.30
sis		9	75	354		562		5	34	56
	Standard	0.841	0.70	1.8	1.0236	1.56	0.3021	0.059	0.22	0.14
	deviation	36	24	765		21		8	75	65
	LLC inspection	−28.29 45	−40. 395 4	−20. 035 5	−59.98 56	−49. 8959	−129.000	−9.84 62	−5.8 652	−8.0 625
Statio narity test	*P* value	0.000								
	ADF-Fish er test	140.9 856	146. 865 2	160 .03 54	74.165 3	1884 .865 2	246.9556	140.0 357	135. 0756	62.0 654
	*P* value	0.000								
	Smoothne	Stable	Stab	Sta	Stable	Stab	Stable	Stabl	Stab	Stab
	ss		le	ble		le		e	le	le

In Table 3, different variables have very small standard deviations, indicating minimal differences between the variables and the overall model. In the results of the stationarity test, this paper utilizes the Levin-Lin-Chu test (LLC) and the ADF-Fisher test, and the *P* of all variables are 0.000, proving the absence of unit roots. To select a suitable model for analyzing the influence of DED on the CE of the research area, this study uses the Hausman test for selection, and the results show *P* = 0.000, indicating the choice of a fixed effects model for analysis. Thus, the regression results can be obtained. To ensure the accuracy, this study uses variable replacement method to conduct test (Table 4).

**Table 4:** Regression results and robustness results.

/	Regression results		Robustness check			
	(1) ^ln *CO*2^	(2) ^ln *CO*2^	Replace core explanatory variables		Replace the explained variable	
			(1) ^ln *CO*2^	(2) ^ln *CO*2^	(1) ln_ *A* _ *CO*_2_	(2) ln_ *A* _ *CO*_2_
ln *EC*	/	0.8812 (6.58 42)^~^	/	0.7986 (5.43 28)^~^	/	0.7982 (6.02 45)^~^
*D*	−0.0705 (−2.1 032)^#^	−0.0439 (−2.5 137)^#^	−1.4512 (−3. 8952)^~^	−0.3712 (−1.1 395)^*^	−0.0617 (−2.0158)^#^	−0.0367 (−2.0265)^#^
ln *TPR*	/	1.8126 (2.90 16)^~^	/	1.3598 (1.9862)^*^	/	1.2501 (2.0645)^*^
*TI*	/	0.0517 (3.36 75)	/	0.0015 (0.0258)	/	0.0485 (0.88475)
^ln^ (^*PC* −^ ^*GDP*^)	/	0.2601 (0.8498)	/	0.3220 (0.95 17)	/	0.1958 (0.59784)
*UL*	/	−4.9956 (−3.3324)^~^	/	−5.3946 (−3.2 567)^~^	/	−6.1624 (−4.0 156)^~^
*IS*	/	0.5496 (3.36 75)^~^	/	0.498 5(2.86 94)^~^	/	0.5402 (3.15 93)^~^
*RSP*	/	0.2435 (1.90 14)^*^	/	0.1523 (1.07 84)	/	0.1726 (1.25 15)
_cons	6.3985 (155.1359)^~^	−16.7985 (−2.6013)^#^	10.4062(5.7 315)^~^	−13.9585(−1.9562)^#^	3.2618(80.0 682)^~^	−14.1278 (−2.0568)^#^
Individual effect	Y	Y	Y	Y	Y	Y
Time effect	Y	Y	Y	Y	Y	Y

Note: The data in parentheses are t-statistics, with “*”, “#”, and “~” indicating significance at the 10%, 5%, and 1% levels. Y = Yes.

In Table 4, for every unit increase in DED level in the study area, the corresponding decrease in CE amount is 0.0439 units. The four indicators of EC, TPR, IS, and RSP can all have a positive promoting effect on CE, while the other indicators have an inhibitory effect on CE levels. The above results may be due to the well-developed urbanization level in the study area, which can effectively control the growth rate of CE volume. The consistency between the robustness results and the regression results indicates that the analysis of the impact on CE mentioned above is reliable, and the constructed model also has excellent stability.

### 2.3 Performance analysis of prediction based on PSO-BP model

**Fig 9:**
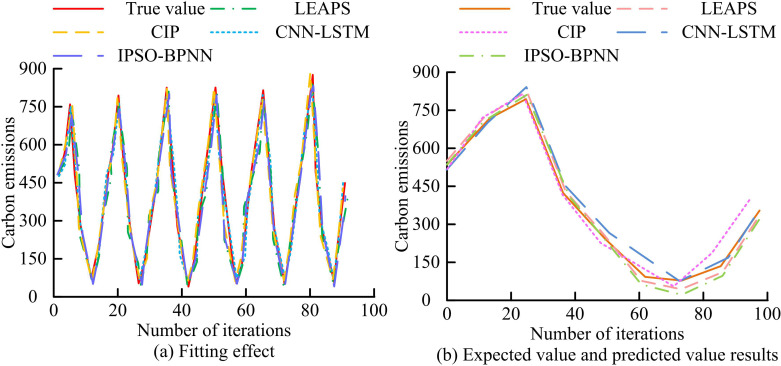
Comparison of training effectiveness of different CE prediction methods.

To validate the CIP’s predictive performance, this study conducted comparative experiments using current mainstream CE prediction methods, namely CNN-LSTM, Long range Energy Alternatives Planning System (LEAPS) based on scenario analysis, and Improved PSO-BP Neural Network (IPSO-BPNN). The evaluation indicators selected were mainstream Mean Absolute Error (MAE), Root MSE (RMSE), Goodness of Fit (GF), and Mean Square Error (MSE). GF is an indicator used in statistics to measure the fit of statistical models to observed data, reflecting the consistency between model predictions and actual observations. MATLAB was chosen as the experimental platform. The amount of neurons in the input, hidden, and output layers of the experimental parameters are set to 8, 12, and 1, respectively. The learning rate and Min error are 0.01 and 0.001. The population size and learning factor are 200 and 2. $\omega_{\max}$ = 0.9 and $\omega_{\min}$ = 0.5. The crossover and mutation probabilities are 0.4 and 0.05. The samples are segmented into a training set and a testing set in an 8:1 ratio. Fig 9 compares the training effectiveness of different CE prediction methods.

Fig 9 (a) shows the fitting effects of different CE prediction methods. The fitting values of the CIP model have a high degree of overlap with the true values, indicating a good fitting effect. The fitting value variation curve of CNN-LSTM shows significant deviations multiple times, while the fitting value variation curves of the other two methods have smaller deviations. Fig 9 shows the predicted values and expected values of different CE prediction methods. CNN-LSTM and IPSO-BPNN have a significant amount of data deviation from expected values, resulting in average prediction performance, while the CIP model only has a few data points slightly deviating from expected values. To further evaluate the performance of various CE prediction methods, various evaluation indicators are used for analysis, as exhibited in Table 5.

**Table 5:** Evaluation results based on different carbon emission prediction methods.

Method	Number	MAE	RMSE	GF	MSE
CIP	1	16.985	21.462	0.9937	516.465
	2	17.064	20.546	0.9936	517.063
	3	16.864	22.059	0.9936	516.954
	Mean value	16.971	21.356	0.9936	516.827
IPSO-BPNN	1	59.945	77.514	0.9356	6135.654
	2	60.055	78.054	0.9247	6114.598
	3	59.846	77.945	0.9324	6127.549
	Mean value	59.949	77.838	0.931	61525.934
LEAPS	1	62.546	80.652	0.915	7264.856
	2	63.421	81.516	0.907	7156.621
	3	62.945	81.065	0.911	7198.546
	Mean value	62.971	81.078	0.911	7206.674
CNN-LSTM	1	73.621	90.132	0.897	8136.462
	2	73.169	89.846	0.904	8101.561
	3	74.063	90.064	0.899	8124.056
	Mean value	73.618	90.010	0.900	8120.693

**Fig 10:**
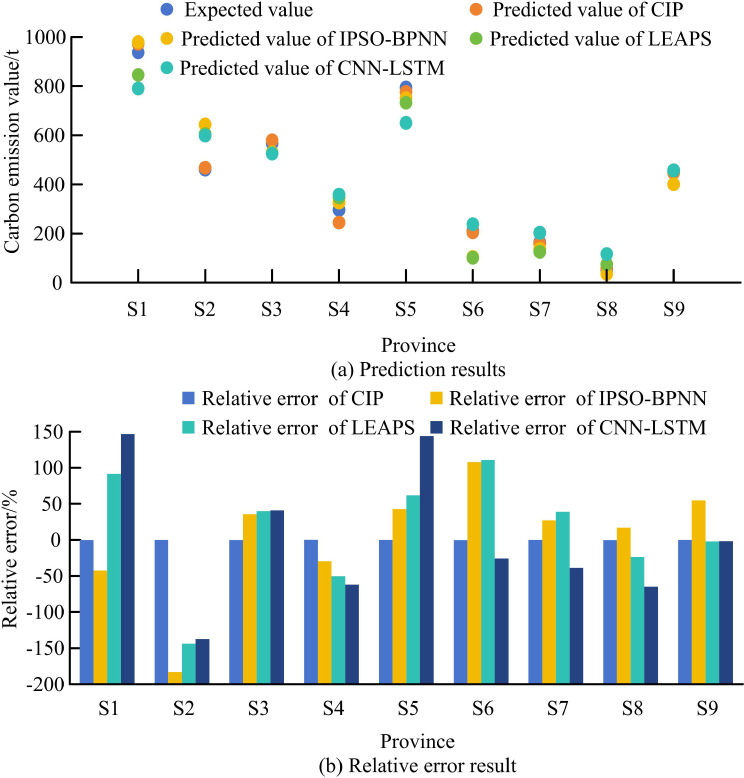
Predicted carbon emissions for each province in the study area.

In Table 5, the fitting effect and performance of the CIP are superior to other prediction methods, with corresponding average MAE, average RMSE, average GF, and average MSE of 16.971, 21.357, 0.9936, and 516.827, respectively. At the same time, the GF values of all methods exceed 85%, indicating that each CE prediction method fits the data very closely and the prediction results have high credibility. Finally, this study uses various CE quantity prediction methods to forecast the CE quantities of each province in the research area, as displayed in Fig 10.

Figs 10(a) and 10(b) are the prediction results and relative errors of different CE quantity prediction methods for each province. In the prediction performance of various provinces in the research area in 2023, the relative error of the CIP model is mostly less than 5%, with only the relative error in S4 and S7 provinces not exceeding 20%. The relative error range of other methods is between −200% and 150%. This indicates that the research method has superior predictive performance in practical applications.

## 3 Discussion and conclusion

Developing the DE is a strategic choice to seize the new chances of the new round of industrial transformation. It is beneficial to stimulate the establishment of a new development pattern and constructing new competitive advantages for the country. The advantage of China’s promotion of DED lies in the fact that the added value of core industries in the DE was projected to exceed 12 trillion yuan by 2023, and it has already built the world’s largest, widely covered, and technologically advanced mobile communication network and fiber optic network. However, developing countries still face profound challenges between digitization and environmental sustainability. This requires developing countries to balance environmental protection and ensure the digitalization process while promoting technological growth, without sacrificing the environment. Therefore, this study takes YREB with imbalanced DED levels as the research object to analyze the influencing factors and pathways of CE under DED. A method for measuring the level of DED and CE in the research area was proposed, and finally a CIP model was designed to predict the CE quantity.

The experiment showed that this study extracted three principal components, F1 ~ F3. In F1, indicators such as E12, E15, E17, and E21 had the highest number of loads. In F2, E51, E52, E53 and other indicators had the highest number of loads. In F3, E16 and E32 had the highest number of loads. The principal components F1 ~ F3 were respectively named as digital ecological environment, digital finance, and digital industrialization. In the final indicator weight results determined by the mixed method, Y1E1 had the highest weight value (33.845%), followed by Y2E2 (19.42%), and Y3E1 has the lowest weight value (12.275%). The above results indicate that the foundation of regional DED level depends on infrastructure, and although social digitization has a small weight, it is still an important component of regional DED.

In the fixed effects model analysis of the impact of various indicators in the study area on CE under DED, for every unit increase in DED level, the corresponding CE amount decreased by 0.0439 units. The four indicators of EC, TPR, IS, and RSP could all have a positive promoting effect on CE, while the other indicators have an inhibitory effect on CE levels. This may be because the urbanization level in the study area is relatively well-developed, which can effectively control the growth rate of CE volume. In addition, the robustness results are correlated with the regression results, showing that the analysis of the impact on CE mentioned above is reliable, and the constructed model also has excellent stability.

In the CIP prediction experiment, the overall fitting effect and performance of the CIP model were superior to other prediction methods, with corresponding average MAE values, average RMSE values, average GF values, and average MSE values of 16.971, 21.357, 0.9936, and 516.827. At the same time, the GF values of all methods exceeded 85%, indicating that each CE prediction method fits the data very closely and the prediction results have high credibility. In practical applications, the relative error of research methods was mostly less than 5%, with only the relative error in S4 and S7 provinces not exceeding 20%. The relative error range of other methods was between -200% and 150%. This confirms the superiority of the research method in application.

Based on the above results analysis, we can delve into how to address the uneven development of the Ganshan digital economy and strategies to suppress carbon emissions in practical applications in the research area. Specifically, it is necessary to first combine the development status of the research region, promote the development of the digital economy according to local conditions, and encourage regions with relatively fast levels of digital economy development to drive regions with slower development, achieving regional coordinated development. Then it is necessary to seize the opportunities of digital development, increase investment in digital infrastructure, develop digital industries, and promote the transformation of traditional industries towards green and low-carbon industries; At the same time, we will promote the integration of urban and rural areas to achieve balanced development and vigorously unleash the vitality of the digital economy. Finally, increase education investment in the research area, improve the overall quality of residents, promote green development in the region, optimize the education structure, strengthen the education level in economically underdeveloped areas, and narrow the gap in human capital between regions. In addition, energy, as a fundamental industry, is also accelerating its transformation towards green, low-carbon, digital, and intelligent development driven by the fourth technological revolution. However, it still needs to face some problems. Firstly, the investment in energy innovation continues to grow, but the intensity of investment is still lower than the national average and that of technology powerhouses such as the United States. Secondly, the output level of energy innovation has significantly increased, and some digital technologies or intelligent devices have been localized and replaced. However, there are still “bottlenecks” in key core technologies. Thirdly, there are significant differences in digital transformation between regions and segmented industries, and the coupling between energy technology and digital technology needs to be improved. The digital transformation of energy needs to address the coordinated integration of energy technology and digital technology, and steadily improve the efficiency of energy production, transportation, distribution, and management on the basis of ensuring energy security through technological integration, while reducing the carbon emission intensity and total amount of economic activities. To address the practical application issues mentioned above, the following solutions can be proposed: firstly, to comprehensively increase the technological innovation and research and development investment of various segmented energy product manufacturers, and form a dynamic mechanism of innovation driven high-quality energy development. The second is to focus on key “bottleneck” technologies in various segmented industries, achieve independent and controllable energy technology, and improve the construction of independent innovation system in the energy field. The third is to promote the digital transformation of the power system, attach importance to the management of digital resources in the energy industry, and ensure the security of energy data.

In summary, the research deeply analyzes the reasons for the imbalance and backwardness of regional DED levels, and provides diagnostic control strategies to suppress CE as a starting point, achieving a win-win situation for ecological environment and economy. However, this study still has certain limitations, such as only analyzing the influencing factors and predictions of regional DED levels and CE from a provincial perspective. In future research, more detailed regional divisions such as city level, county level, and village level can be explored to obtain more detailed results.

## Supporting Information

S1 file Minimal data set. (DOCX)
