# Supplementary material for: Factors and prediction of carbon emissions based on PSO-BP neural network model under the development of digital economy
Source: PLoS One. 2025 May 8;20(5):e0322703. doi: 10.1371/journal.pone.0322703 (PMC12061098; doi:10.1371/journal.pone.0322703)
Supplement: S1 file — (DOCX) [file pone.0322703.s001.docx]

**The data in Figure 5**

| Third level indicators | F1 | F2 | F3 |
| --- | --- | --- | --- |
| E11 | 0.025 | -0.010 | 0.000 |
| E12 | 0.031 | 0.003 | -0.012 |
| E13 | 0.029 | 0.013 | -0.019 |
| E14 | 0.030 | -0.011 | -0.012 |
| E15 | 0.030 | -0.004 | -0.014 |
| E16 | 0.001 | 0.051 | 0.031 |
| E17 | 0.029 | -0.011 | -0.005 |
| E21 | 0.002 | 0.019 | 0.098 |
| E22 | 0.027 | -0.006 | 0.022 |
| E23 | 0.029 | -0.020 | 0.004 |
| E31 | 0.031 | -0.012 | 0.001 |
| E32 | 0.030 | -0.005 | 0.007 |
| E33 | 0.029 | -0.004 | 0.009 |
| E41 | 0.030 | -0.010 | 0.013 |
| E42 | 0.030 | -0.012 | 0.016 |
| E43 | 0.029 | -0.015 | 0.015 |
| E51 | 0.012 | 0.057 | -0.002 |
| E52 | 0.017 | 0.048 | -0.002 |
| E53 | 0.017 | 0.050 | -0.015 |
| E54 | 0.009 | 0.041 | -0.029 |

**The data in Figure 6**

| Third level indicators | GPCA method | EM method | Mixed method |
| --- | --- | --- | --- |
| E11 | 5.91 | 2.90 | 4.40 |
| E12 | 5.41 | 6.66 | 6.03 |
| E13 | 5.28 | 3.21 | 4.24 |
| E14 | 5.61 | 4.06 | 4.83 |
| E15 | 6.04 | 4.27 | 5.15 |
| E16 | 3.30 | 2.20 | 2.75 |
| E17 | 6.15 | 4.69 | 5.42 |
| E21 | 2.38 | 12.48 | 7.43 |
| E22 | 4.68 | 10.38 | 7.53 |
| E23 | 4.54 | 3.77 | 4.15 |
| E31 | 5.15 | 5.50 | 5.32 |
| E32 | 4.86 | 3.98 | 4.42 |
| E33 | 4.20 | 5.27 | 4.73 |
| E41 | 6.56 | 8.27 | 7.41 |
| E42 | 6.53 | 5.12 | 5.82 |
| E43 | 4.86 | 7.50 | 6.18 |
| E51 | 6.28 | 1.18 | 3.73 |
| E52 | 5.08 | 1.85 | 3.46 |
| E53 | 4.95 | 0.95 | 2.95 |
| E54 | 3.01 | 1.25 | 2.13 |

**The data in Figure 7**

| - | 2016 | 2017 | 2018 | 2019 | 2020 | 2021 | 2022 | 2023 |
| --- | --- | --- | --- | --- | --- | --- | --- | --- |
| S1 | 6.19 | 7.18 | 7.8 | 7.79 | 8.61 | 9.35 | 10.12 | 11.75 |
| S2 | 3.6 | 4.24 | 4.66 | 5.23 | 5.53 | 5.76 | 6.02 | 6.35 |
| S3 | 1.62 | 1.95 | 2.17 | 2.48 | 2.7 | 2.81 | 2.96 | 3.02 |
| S4 | 2.36 | 2.79 | 3.19 | 3.68 | 3.89 | 4.07 | 4.25 | 5.01 |
| S5 | 1.45 | 1.72 | 1.81 | 2.05 | 2.21 | 2.36 | 2.47 | 2.85 |
| S6 | 0.73 | 0.97 | 1.11 | 1.91 | 1.28 | 1.36 | 1.53 | 2.04 |
| S7 | 1.08 | 1.36 | 1.54 | 1.74 | 1.86 | 1.21 | 1.35 | 1.96 |
| S8 | 0.6 | 0.8 | 0.94 | 1.05 | 1.15 | 1.26 | 1.43 | 1.98 |
| S9 | 1.21 | 1.41 | 1.71 | 1.86 | 2.02 | 2.27 | 2.53 | 2.79 |

**The data in Figure 8**

| - | 2016 | 2017 | 2018 | 2019 | 2020 | 2021 | 2022 | 2023 |
| --- | --- | --- | --- | --- | --- | --- | --- | --- |
| S1 | 8.33 | 8.06 | 9.0165 | 9.3712 | 9.1226 | 8.8752 | 8.6554 | 8.7526 |
| S2 | 5.13 | 4.94 | 4.9068 | 4.6063 | 5.0264 | 4.8562 | 4.5625 | 4.7652 |
| S3 | 4.49 | 4.88 | 5.418 | 5.6648 | 5.9326 | 6.1253 | 5.8361 | 5.7521 |
| S4 | 2.65 | 2.62 | 2.7617 | 2.9627 | 2.7416 | 2.6542 | 2.6325 | 2.7526 |
| S5 | 5.9 | 6.39 | 7.2357 | 7.9428 | 6.4142 | 6.223 | 6.1215 | 6.0213 |
| S6 | 1.37 | 1.75 | 1.99159 | 2.1241 | 2.0136 | 1.8532 | 1.9523 | 1.9852 |
| S7 | 1.52 | 1.51 | 1.6299 | 1.6449 | 1.6352 | 1.6215 | 1.6023 | 1.6132 |
| S8 | 0.56 | 0.53 | 0.5194 | 0.5175 | 0.5125 | 0.5023 | 0.5075 | 0.5103 |
| S9 | 3.7 | 4.04 | 4.2154 | 4.5528 | 4.5652 | 4.5598 | 4.5512 | 4.5585 |

**The data in Figure 9**

| - | 2016 | 2017 | 2018 | 2019 | 2020 | 2021 | 2022 | 2023 |
| --- | --- | --- | --- | --- | --- | --- | --- | --- |
| S1 | 8.35 | 8.03 | 8.95 | 9.27 | 9.02 | 8.97 | 9.00 | 9.15 |
| S2 | 5.25 | 5.03 | 4.97 | 4.65 | 4.63 | 4.58 | 4.56 | 4.61 |
| S3 | 12.78 | 13.90 | 15.47 | 16.20 | 15.38 | 15.12 | 15.43 | 15.56 |
| S4 | 6.84 | 6.71 | 7.03 | 7.51 | 7.47 | 7.32 | 7.35 | 7.43 |
| S5 | 24.22 | 26.26 | 29.87 | 32.89 | 30.12 | 29.86 | 29.75 | 30.25 |
| S6 | 19.71 | 24.82 | 26.98 | 29.63 | 29.52 | 29.46 | 29.50 | 29.71 |
| S7 | 6.03 | 5.99 | 6.48 | 6.56 | 6.49 | 6.39 | 6.42 | 6.53 |
| S8 | 9.62 | 9.04 | 8.85 | 8.77 | 8.74 | 8.68 | 8.72 | 8.82 |
| S9 | 15.24 | 16.29 | 16.73 | 17.79 | 16.98 | 16.85 | 16.90 | 17.53 |

**The data in Figure 10**

| Evaluation | Method | 0 | 20 | 40 | 60 | 80 |
| --- | --- | --- | --- | --- | --- | --- |
| Fitting effect | True value | 465 | 753 | 276 | 309 | 862 |
|  | LEAPS | 464 | 750 | 271 | 313 | 764 |
|  | CIP | 462 | 748 | 273 | 307 | 758 |
|  | CNN-LSTM | 461 | 752 | 275 | 311 | 843 |
|  | IPSO-BPNN | 465 | 751 | 276 | 310 | 859 |
| Expected value and predicted value results | True value | 533 | 762 | 379 | 93 | 106 |
|  | LEAPS | 559 | 770 | 386 | 82 | 86 |
|  | CIP | 521 | 771 | 374 | 102 | 115 |
|  | CNN-LSTM | 519 | 772 | 391 | 127 | 109 |
|  | IPSO-BPNN | 532 | 764 | 381 | 80 | 84 |

**The data in Figure 11**

| Evaluation | Method | S1 | S2 | S3 | S4 | S5 | S6 | S7 | S8 | S9 |
| --- | --- | --- | --- | --- | --- | --- | --- | --- | --- | --- |
| Predicted value | Expected value | 937.120 | 460.630 | 566.480 | 296.270 | 794.280 | 212.410 | 164.490 | 51.750 | 455.280 |
|  | CIP | 972.118 | 467.596 | 579.461 | 244.522 | 776.367 | 205.237 | 159.100 | 60.362 | 447.428 |
|  | IPSO-BPNN | 979.509 | 643.968 | 530.816 | 325.996 | 751.395 | 104.540 | 137.381 | 34.984 | 400.658 |
|  | LEAPS | 845.625 | 604.627 | 526.462 | 346.595 | 732.515 | 101.652 | 125.462 | 75.621 | 457.265 |
|  | CNN-LSTM | 790.421 | 598.197 | 525.417 | 358.443 | 650.240 | 238.363 | 203.106 | 116.698 | 456.970 |
| Relative error | CIP | -0.037 | -0.015 | -0.023 | 0.175 | 0.023 | -0.166 | 0.033 | -0.166 | 0.017 |
|  | IPSO-BPNN | -42.389 | -183.338 | 35.664 | -29.726 | 42.885 | 107.870 | 27.109 | 16.766 | 54.622 |
|  | LEAPS | 91.495 | -143.997 | 40.018 | -50.325 | 61.765 | 110.758 | 39.028 | -23.871 | -1.985 |
|  | CNN-LSTM | 146.699 | -137.567 | 41.063 | -62.173 | 144.040 | -25.953 | -38.616 | -64.948 | -1.690 |
